# Intergenerational Impact of Parental Zinc Deficiency on Metabolic and Redox Outcomes in *Drosophila melanogaster*

**DOI:** 10.3390/biology13060401

**Published:** 2024-06-01

**Authors:** Kamaldeen Olalekan Sanusi, Kasimu Ghandi Ibrahim, Murtala Bello Abubakar, Tijjani Salihu Shinkafi, Aminu Ishaka, Mustapha Umar Imam

**Affiliations:** 1 Centre for Advanced Medical Research and Training, Usmanu Danfodiyo University Sokoto, Sokoto P.M.B. 2346, Nigeria; sanusikamaldeen@yahoo.com; 2 Department of Physiology, Faculty of Basic Medical Sciences, College of Health Sciences, Usmanu Danfodiyo University Sokoto, Sokoto P.M.B. 2346, Nigeria; 3 Department of Human Physiology, Faculty of Health Sciences, Al-Hikmah University, Ilorin P.M.B. 1601, Nigeria; 4 Department of Basic Medical and Dental Sciences, Faculty of Dentistry, Zarqa University, P.O. Box 2000, Zarqa 13110, Jordan; kibrahim@zu.edu.jo; 5 Department of Physiology, College of Medicine and Health Sciences, Sultan Qaboos University, Muscat 123, Oman; m.abubakar@squ.edu.om; 6 Department of Biochemistry and Molecular Biology, Faculty of Chemical and Life Sciences, College of Health Sciences, Usmanu Danfodiyo University Sokoto, Sokoto P.M.B. 2346, Nigeria; tijjani.salihu@udusok.edu.ng; 7 Department of Biochemistry, Kampala International University, Western Campus, Bushenyi P.O. Box 71, Uganda; 8 Department of Medical Biochemistry, Faculty of Basic Medical Sciences, College of Health Sciences, Usmanu Danfodiyo University Sokoto, Sokoto P.M.B. 2346, Nigeria; ishaka.aminu@udusok.edu.ng; 9 Department of Medical Biochemistry, Faculty of Basic Medial Sciences, College of Health Sciences, Nile University of Nigeria, Abuja F.C.T. 900108, Nigeria

**Keywords:** parental zinc deficiency, metabolic pathways, intergenerational effects, *Drosophila melanogaster*, zinc transport

## Abstract

**Simple Summary:**

Zinc is an important nutrient for our bodies, but many people do not get enough of it. This study looked at fruit flies (Drosophila) to see if low zinc levels in both parents could affect their babies’ health. We found that the offspring of zinc-deficient parents gained more weight and had higher zinc levels themselves. However, these offspring also had problems with how their bodies used sugar and fat, and their ability to fight off damage from the environment was reduced. Interestingly, the study suggests these effects were worse in female offspring. This research highlights the importance of zinc for overall health and suggests that low zinc levels in parents might have consequences for their children. Further studies on these connections can help us develop strategies to improve health for future generations.

**Abstract:**

Zinc deficiency is a common nutritional disorder with detrimental health consequences. Whether parental zinc deficiency induces intergenerational effects remains largely unknown. We investigated the effects of a combined maternal and paternal zinc deficiency on offspring’s metabolic outcomes and gene expression changes in *Drosophila melanogaster*. The parent flies were raised on zinc-deficient diets throughout development, and their progeny were assessed. Offspring from zinc-deprived parents exhibited a significant (*p* < 0.05) increase in body weight and whole-body zinc levels. They also displayed disrupted glucose metabolism, altered lipid homeostasis, and diminished activity of antioxidant enzymes. Gene expression analysis revealed significant (*p* < 0.05) alterations in zinc transport genes, with increases in mRNA levels of *dZIP1* and *dZnT1* for female and male offspring, respectively. Both sexes exhibited reduced *dZnT35C* mRNA levels and significant (*p* < 0.05) increases in the mRNA levels of *DILP2* and proinflammatory markers, *Eiger* and *UPD2.* Overall, female offspring showed higher sensitivity to parental zinc deficiency. Our findings underscore zinc’s crucial role in maintaining health and the gender-specific responses to zinc deficiency. There is the need for further exploration of the underlying mechanisms behind these intergenerational effects.

## 1. Introduction

Zinc is a micronutrient integral to diverse physiological processes related to growth and immune system function. Maintaining optimal zinc levels is imperative for overall health, with disruptions linked to metabolic, antioxidant, and inflammatory disorders [1,2]. Zinc deficiency is common among pregnant women and has long been linked to growth and developmental disruptions in their fetuses [3]. This is because sufficient zinc stores are crucial for the rapid growth phase during fetal development, which significantly increases the demands for zinc during pregnancy [4]. Furthermore, maternal zinc restriction during pregnancy has been associated with lower birth weight and altered growth trajectories, resulting in increased body fat and reduced lean mass later in life [5]. Additionally, maternal zinc restriction and its impact on pregnancy outcomes have been associated with reduced production of essential zinc-related enzymes and proteins required for growth and development [6].

However, understanding the impact of zinc deficiency on offspring health requires a comprehensive approach that goes beyond studying maternal zinc deficiency alone. While maternal zinc deficiency has been relatively researched and linked to adverse pregnancy outcomes, focusing solely on maternal factors overlooks the potential contributions of paternal lineage and the combined effects of parental zinc deficiency. Both parents contribute genetic material and play unique roles in the developmental trajectory of their offspring. Therefore, investigating the combined impact of maternal and paternal zinc deficiencies allows for more understanding of how parental factors influence the health outcomes of future generations.

To assess the parental zinc deficiency and its far-reaching consequences, this study employs the versatile fruit fly (Drosophila) model. Drosophila offers several advantages for zinc deficiency research including short life cycle, which allows for rapid multi-generational studies; genetic tractability, which enables targeted manipulation of genes related to zinc metabolism; conserved biological pathways whereby many human disease-related pathways are also present in Drosophila, facilitating translation of findings; and well-established methods, whereby extensive research history using Drosophila makes experimental protocols and data interpretation reliable.

Accordingly, this study hypothesizes that a combined maternal and paternal zinc deficiency will have far-reaching consequences for metabolic, antioxidant, and inflammatory pathways in both parent flies and their offspring. We aimed to investigate how parental zinc deficiency impacts various parameters like body weight, zinc levels, glucose metabolism, lipid homeostasis, antioxidant enzyme activity, and the expression of key zinc transporter genes. Additionally, we explored the influence of zinc deficiency on metabolic markers (DILP2 and dPEPCK) and inflammatory factors (EGR and UPD2) to understand potential connections between zinc status and the regulation of critical metabolic pathways.

## 2. Materials and Methods

### 2.1. Fruit Fly (Drosophila melanogaster) Husbandry and Experimental Design

We obtained the W^1118^ strain of *Drosophila melanogaster* from the Fly Laboratory of the Centre for Advanced Medical Research and Training (CAMRET) at Usmanu Danfodiyo University, Sokoto, Nigeria. The flies were cultured and maintained at the optimum temperature range of 22–25 °C, relative humidity between 50 and 60%, and exposed to a natural light–dark cycle. Their diet consisted of a standard cornmeal mixture specifically formulated for Drosophila: corn flour, agar, yeast, methyl paraben, and distilled water. To induce zinc deficiency in the parent generation (F0), the zinc-chelator TPEN (N,N,N′,N′-tetrakis(2-pyridylmethyl)ethylenediamine) was incorporated into their diet at a concentration of 100 μM [7]. Gravid adult flies aged seven to ten days were transferred to the zinc-chelated diet and given a 24 h period to lay eggs. The eggs were allowed to develop on the zinc-chelated diet, and the resulting adults were referred to as the F0 generation. Adult male and virgin female flies (F0) that developed on a zinc-deficient diet were mated on the standard diet to generate F1. The offspring (F1) were maintained on normal diet for seven days and analyzed for physical variables, biochemical variables, and gene expression. To ensure optimal food quality and prevent contamination, fresh media vials were provided weekly throughout the study.

### 2.2. Measurement of Total Body Zinc

The total body zinc levels of the zinc-deficient parent generation (F0) and the offspring (F1) were assessed at adulthood. Three independent groups of ten adult flies, aged 7–10 days, were collected and anesthetized using ice immobilization. Each group was meticulously rinsed with distilled water to remove any external contaminants. For individual analysis, the flies were carefully transferred to sterile microcentrifuge tubes and individually digested in 1 mL of concentrated nitric acid (65% HNO_3_). The tubes were then heated on a block at 100 °C for ten minutes to ensure complete digestion. After cooling, the samples were diluted to 5 mL with distilled water to achieve a consistent concentration suitable for analysis. The total body zinc concentration for each group was measured using an Agilent Microwave Plasma Atomic Emission Spectrometer (MP-AES). Calibration curves ranging from 0.00 to 6.00 ppm zinc were established on the MP-AES to ensure accurate measurements.

### 2.3. Determining Fly Body Weight

To determine the average weight of the flies, groups of ten flies were gently anesthetized by chilling them on ice. From each group, three separate samples were carefully collected and weighed on a sensitive electronic balance (Kern & Sohn Ltd., Balingen, Germany). The weight of each sample was recorded in milligrams, allowing for the accurate calculation of the group’s average weight.

### 2.4. Biochemical Analysis

#### 2.4.1. Sample Preparation

To remove gut contents and prepare for biochemical analysis, flies were first transferred to empty vials for an hour to allow for gut clearance. Subsequently, they were gently anesthetized on ice and rinsed with ice-cold phosphate-buffered saline (PBS, pH 7.4, 1:5 *w*/*v*) to remove external contaminants. Per group, ten (10) flies aged 7–10 days were pooled in three replicates (n = 30 per group). Homogenous pooling was ensured by carefully measuring fly weight before sample preparation. The flies were then homogenized and centrifuged at 3000× *g* for 6 min at 4 °C using a refrigerated centrifuge (MX-301 Highspeed, Tomy Kogyo Co., Ltd., Tokyo, Japan). The supernatant from the whole fly extract were thereafter used for the biochemical analysis [8].

#### 2.4.2. Glucose Assay

In the whole fly extract, glucose levels were determined using a commercially available Spinreact™ kit (Girona, Spain) following the manufacturer’s instructions. Samples and standards were read against a blank at 505 nm using an MPR-H200BC Microplate Reader (Infitek, Jinan, China). The glucose concentration was calculated using the following formula:

Glucose concentration (mg/dL) = (Absorbance of sample/Absorbance of standard) × 100

#### 2.4.3. Trehalose Assay

Trehalose levels were quantified using a colorimetric assay kit (Solarbio Life Science, Beijing, China) according to the manufacturer’s protocol. Sample absorbance was measured at 620 nm using an MPR-H200BC Microplate Reader (Infitek, Jinan, China). The final trehalose concentration was calculated using the following formula:

Trehalose (mg/g sample) = Concentrations from *y*-axis/Fresh weight of the sample

#### 2.4.4. Glycogen Assay

In the whole fly extract, the glycogen level was measured using a colorimetric assay kit (Solarbio Life Science, Beijing, China) as per the manufacturer’s instructions. Absorbance was measured at 620 nm on an MPR-H200BC Microplate Reader (Infitek, Jinan, China). The final concentration was calculated using the following formula:

Glycogen (mg/g fresh weight) = [(0.1 mg/mL × 25 µL) × (A3 − A1)]/[(A2 − A1) × (W × 25 µL/1 mL)] × 1.11

1.11 = conversion factor (glucose to glycogen); Cs = standard concentration (0.1 mg/mL); V1 = sample volume (25 µL); V2 = extraction volume (1 mL); W = sample weight; A1 = blank absorbance; A2 = standard absorbance; and A3 = sample absorbance.

#### 2.4.5. Triglyceride Assay

Triglyceride levels in the whole fly extract were quantified using a colorimetric assay kit (Spinreact, Girona, Spain) according to the manufacturer’s protocol. Sample and standard absorbance were read at 505 nm against a blank using an MPR-H200BC Microplate Reader (Infitek, Jinan, China). Triglyceride concentration was calculated as follows:

Triglycerides (mg/dl) = [A(Sample) − A(Blank)]/[A(Standard) − A(Blank)] × 100

#### 2.4.6. Catalase (CAT) Assay

CAT activity was detected using a colorimetric assay kit (Solarbio Life Science, Beijing, China) following the manufacturer’s instructions. The principle relies on CAT decomposing H_2_O_2_ into H_2_O and O_2_, measured at 240 nm (MPR-H200BC Microplate Reader, Infitek, Jinan, China). Activity was calculated using the formula:

CAT activity (U/mL) = [(ΔA × Extraction volume)/(Ɛ × d × 10^9^)]/(Sample volume × Reaction time)

Ɛ = molar coefficient; d = light path length.

#### 2.4.7. Total Antioxidant Capacity (TAOC) Assay

The total antioxidant capacity of samples was measured using a colorimetric assay kit from Solarbio Life Science (Beijing, China) as per the manufacturer’s instructions. This kit quantifies the combined antioxidant potential of molecules and enzymes. It utilizes Fe^3+^-TPTZ, which is transformed into the blue-colored Fe^2+^-TPTZ by antioxidants. The intensity of this blue color, measured at 505 nm (MPR-H200BC Microplate Reader, Infitek, Jinan, China), reflects the total antioxidant capacity. The final concentration was obtained from the following formula:

Total antioxidant capacity (µmol/mL) = x × Vrv ÷ Vs = 34 × x

Vrv = total reaction volume (1.02 mL); Vs = sample volume (0.03 mL); and x = concentration from the standard curve.

#### 2.4.8. Malondialdehyde (MDA) Assay

MDA levels were determined using a lipid peroxidation assay kit (Solarbio Life Science, Beijing, China) according to the manufacturer’s protocol. The final concentration was calculated using the following formula:

MDA (nmol/g) = 5 [6.45 × (ΔA532 − ΔA600) − 1.29 × ΔA450]/sample weight

### 2.5. Gene Expression Analysis

#### 2.5.1. RNA Extraction

RNA was extracted from 15 flies (aged 7–10 days) per group in triplicate (n = 45 per group) using a nucleic acid isolation kit from Hunan Runmei Gene Technology Co., Ltd., Changsha, China, following their established protocol. The extracted RNA’s purity was then assessed using the Bioevopeak Nucleic Acid Analyzer (SP-MUV2000F, Jinan, China). Only samples exhibiting A260/230 and A260/280 ratios within the range of 1.8 to 2.2 were deemed acceptable for further analysis, ensuring high-quality RNA for reliable gene expression measurements.

#### 2.5.2. Primer Design

Specific primers (Table 1) for the genes of interest were designed using the PrimerQuest tool, which incorporates Primer3 software (version 2.2.3) (https://www.idtdna.com/PrimerQuest/Home/Index) assessed on 7 January 2022. RPL32 served as the reference gene, providing a stable baseline for normalization.

#### 2.5.3. Reverse Transcription Quantitative Polymerase Chain Reaction (RT-qPCR) Analysis

For RT-qPCR analysis, we used the TransScript Green One-Step qRT-PCR SuperMix (AQ211) kit from TransGen Biotech Co., Ltd. (Beijing, China), according to the manufacturer’s instructions. Each reaction mixture contained 200 ng/µL of RNA template, 0.4 µL each of forward and reverse primers (100 µM concentration), 10 µL of SuperMix, 0.4 µL of enzyme mix, and RNase-free water, bringing the final volume to 20 µL. The prepared mixtures were then loaded onto a Rotor-Gene Q-5plex HRM platform thermal cycler (Qiagen, Hilden, Germany) and subjected to the cycling conditions on Table 1. The fold change in gene expression was calculated using the formula 2^−ΔΔCT^. The ΔΔCT represents the difference in CT values between the target gene and the reference gene, normalized to the difference between control and treatment groups. This method allows for a relative quantification of gene expression changes between different samples.

### 2.6. Statistical Analysis

Statistical analysis was performed using GraphPad Prism 9.5.1.733 (GraphPad Software Inc., San Diego, CA, USA). We employed a two-way analysis of variance (ANOVA) followed by a Bonferroni’s multiple comparison post hoc test to identify specific group differences. The results were presented as mean ± standard deviation (SD) with a significance level at *p* < 0.05.

## 3. Results

### 3.1. Effects of Parental Zinc Deficiency on Zinc Levels and Body Weights

**Figure 1:**
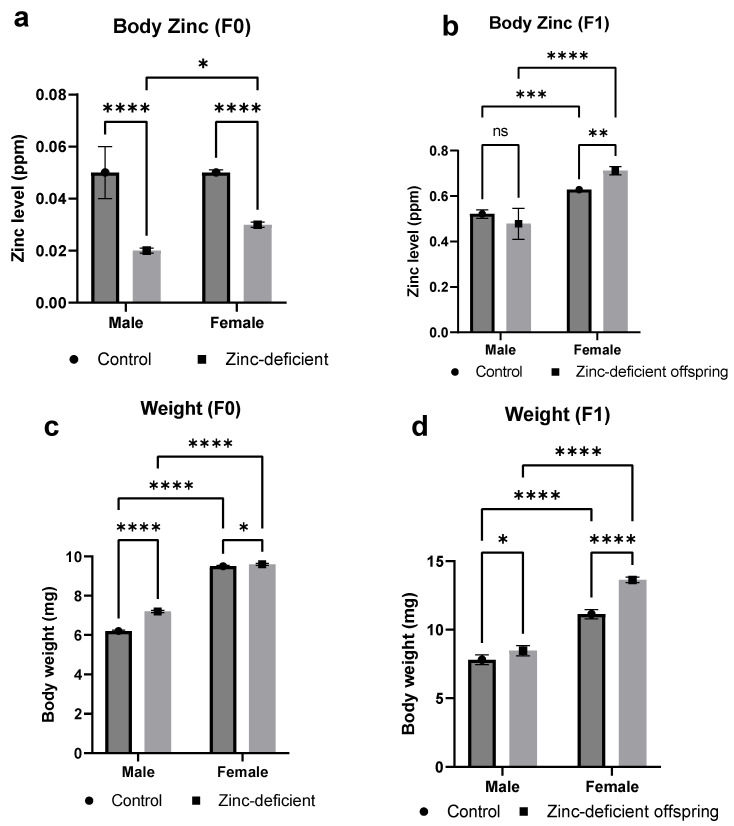
Effects of parental zinc deficiency on zinc levels (**a**,**b**) and body weights (**c**,**d**) of flies. Bars represent mean ± SD. Data were analyzed using two-way ANOVA followed by Bonferroni’s post hoc test. ns: not significant. Asterisks represent significant difference at varying *p* values (*: 0.0332, **: 0.0021, ***: 0.0002, ****: <0.0001, ns: not significant). n = 30 per group.

In comparison with the control group, zinc levels were significantly (*p* < 0.05) reduced in the male and female parents following zinc chelation (Figure 1a). Although no significant difference was observed between the male offspring of zinc-deficient and control groups, a significant increase (*p* < 0.05) was observed in the female offspring compared to control (Figure 1b). There was also a significant (*p* < 0.05) increase in the body weights of the male and female parents and offspring of the zinc-deficient groups compared to control (Figure 1c,d).

### 3.2. Effects of Parental Zinc Deficiency on Glucose and Trehalose Levels

**Figure 2:**
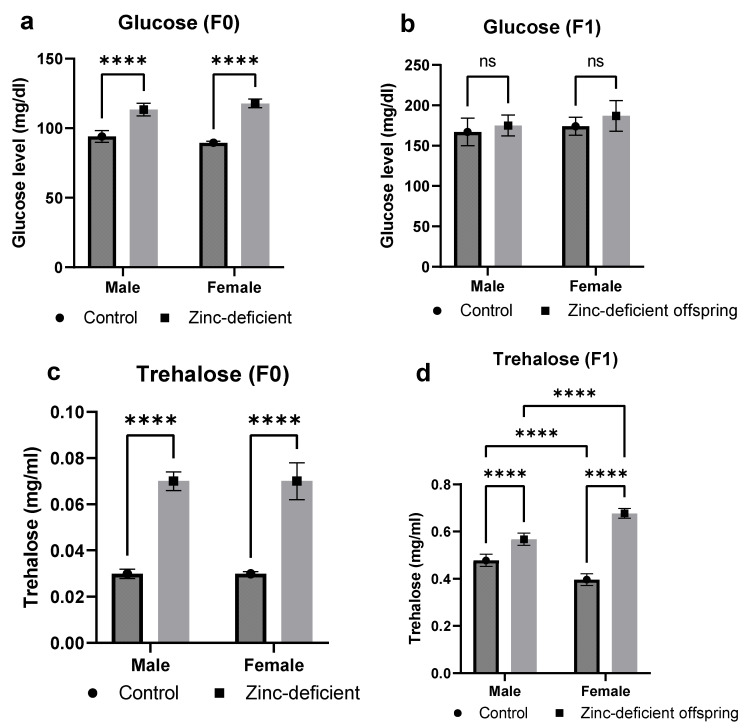
Effects of parental zinc deficiency on glucose (**a**,**b**) and trehalose (**c**,**d**) levels of flies. Bars represent mean ± SD. Data were analyzed using two-way ANOVA followed by Bonferroni’s post hoc test. ns: not significant. Asterisks represent significant difference at varying *p* values (****: <0.0001, ns: not significant). n = 30 per group.

Glucose levels were significantly (*p* < 0.05) increased in the male and female parents (Figure 2a), but no significant difference in glucose levels of the offspring was observed compared to control (Figure 2b). Moreover, both male and female parents and offspring exhibited a significant (*p* < 0.05) increase in trehalose levels compared to control (Figure 2c,d).

### 3.3. Effects of Parental Zinc Deficiency on Glycogen and Triglyceride Levels

**Figure 3:**
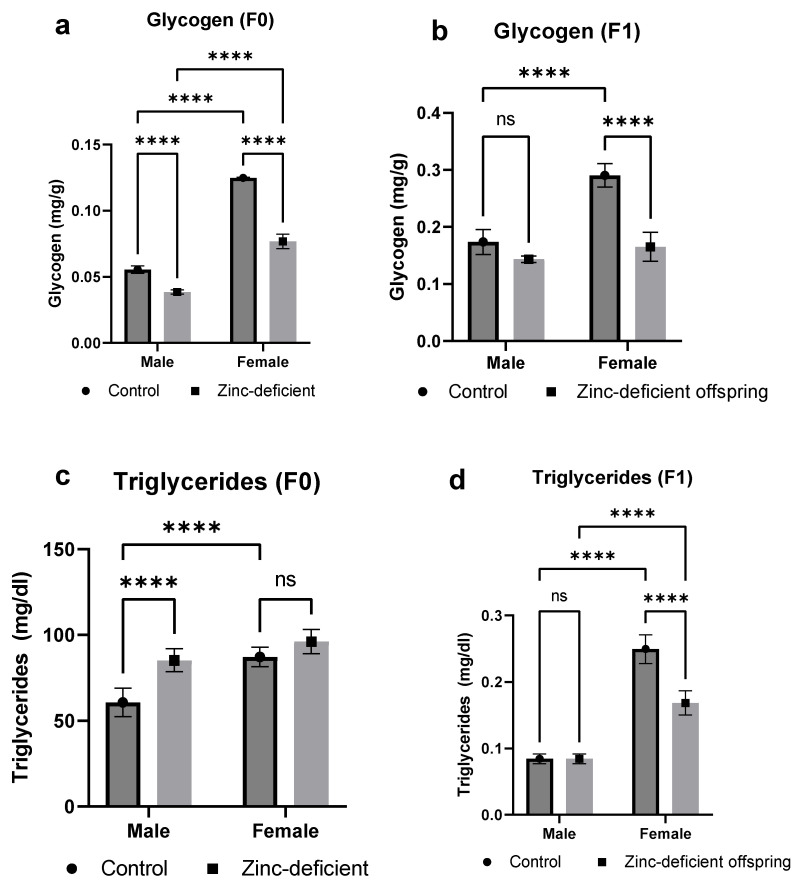
Effect of parental zinc deficiency on glycogen (**a**,**b**) and triglycerides (**c**,**d**) levels of flies. Bars represent mean ± SD. Data were analyzed using two-way ANOVA followed by Bonferroni’s post hoc test. ns: not significant. Asterisks represent significant difference at varying *p* values (****: <0.0001, ns: not significant). n = 30 per group.

The glycogen levels in both parents and offspring were significantly (*p* < 0.05) reduced compared to control (Figure 3a,b). Moreover, triglyceride levels were significantly (*p* < 0.05) increased in the male parents (Figure 3c) but significantly reduced (*p* < 0.05) in the female offspring (Figure 3d).

### 3.4. Effects of Parental Zinc Deficiency on Catalase Activities, Total Antioxidant Capacities, and Malondialdehyde Levels

**Figure 4:**
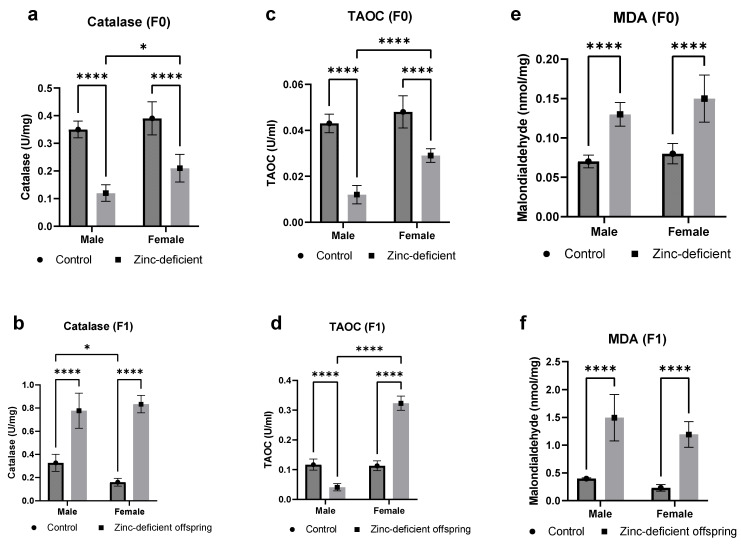
Effects of parental zinc deficiency on catalase activities (**a**,**b**), total antioxidant capacities (**c**,**d**), and malondialdehyde levels (**e**,**f**) of flies. Bars represent mean ± SD. Data were analyzed using two-way ANOVA followed by Bonferroni’s post hoc test. ns: not significant. Asterisks represent significant difference at varying *p* values (*: 0.0332, ****: <0.0001). TAOC: total antioxidant capacity. MDA: malondialdehyde. n = 30 per group.

The zinc-deficient parents showed a significant (*p* < 0.05) reduction in catalase activities (Figure 4a), whereas the male and female offspring had a significant (*p* < 0.05) increase compared to control (Figure 4b). The total antioxidant capacity was significantly reduced in the zinc-deficient parents (Figure 4c). Similarly, the male offspring had a significant reduction while the female offspring had a significant (*p* < 0.05) increase in the total antioxidant capacity (Figure 4d). Moreover, the levels of malondialdehyde were significantly (*p* < 0.05) increased in both parents as well as in the male and female offspring compared to control (Figure 4e,f).

### 3.5. Effects of Parental Zinc Deficiency on mRNA Levels of Zinc Transporters (dZIP1 and dZnT1) Involved in Zinc Absorption

**Figure 5:**
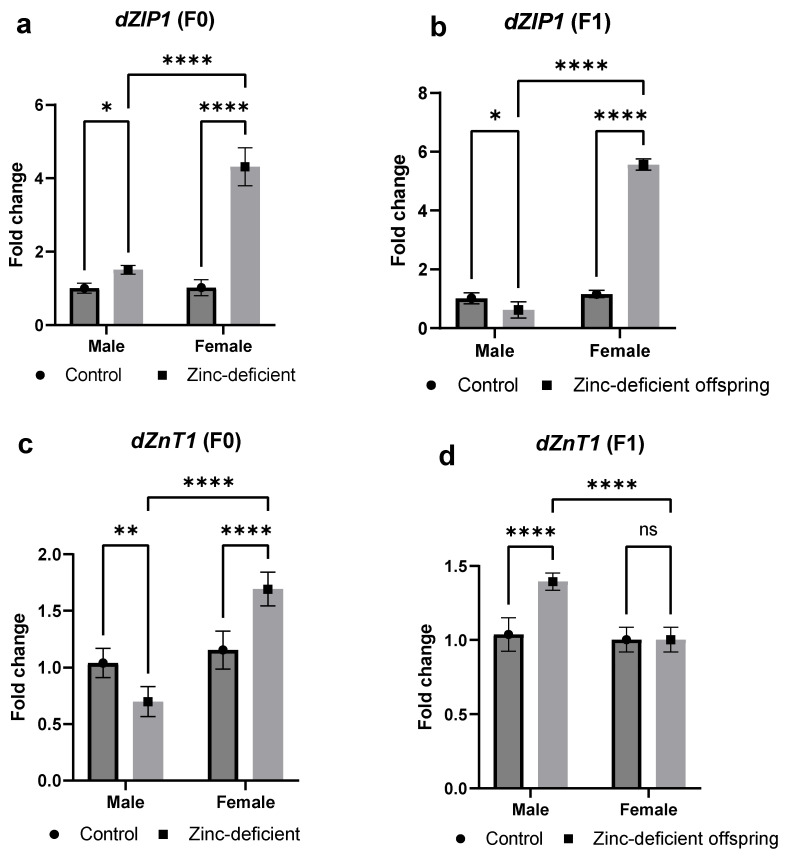
Effects of parental zinc deficiency on *dZIP1* (**a**,**b**) and *dZnT1* (**c**,**d**) mRNA of flies. Bars represent mean ± SD. Data were analyzed using two-way ANOVA followed by Bonferroni’s post hoc test. ns: not significant. Asterisks represent significant difference at varying *p* values (*: 0.0332, **: 0.0021, ****: <0.0001, ns: not significant). n = 45 per group.

There were significant increases in mRNA levels of *dZIP1* in both male and female parents compared to control (Figure 5a). However, the male offspring had a significant (*p* < 0.05) decrease while the female offspring had a significant (*p* < 0.05) increase compared to control (Figure 5b).

In addition, the mRNA levels of *dZnT1* were significantly (*p* < 0.05) increased in the male parent but decreased in the female parent (Figure 5c). Conversely, the male offspring showed a significant increase in *dZnT1* mRNA while no significant (*p* > 0.05) difference was observed in the female offspring compared to control (Figure 5d).

### 3.6. Effects of Parental Zinc Deficiency on Zinc Transporter Genes (dZIP71B and dZnT35C) Involved in Zinc Excretion

**Figure 6:**
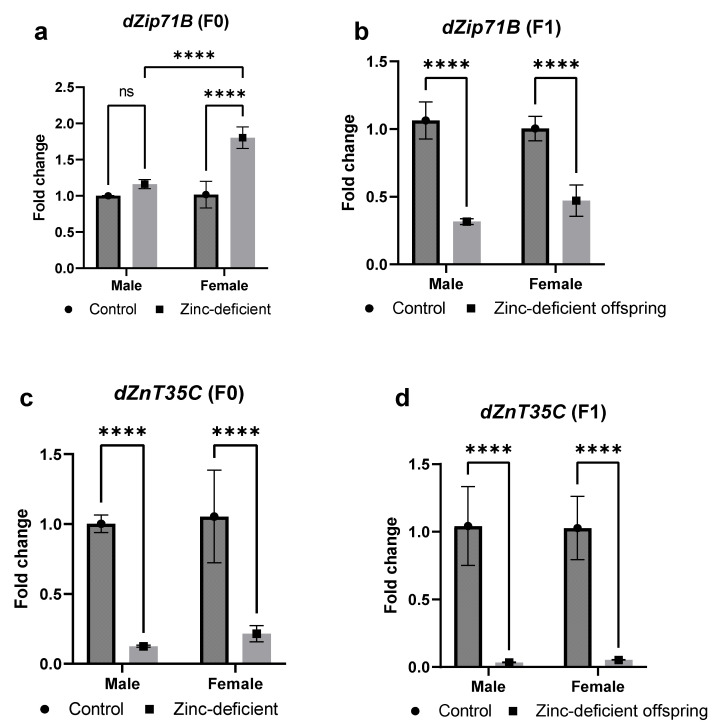
Effects of parental zinc deficiency on *dZip71B* (**a**,**b**) and *dZnT35C* (**c**,**d**) mRNA of flies. Bars represent mean ± SD. Data were analyzed using two-way ANOVA followed by Bonferroni’s post hoc test. ns: not significant. Asterisks represent significant difference at varying *p* values (****: <0.0001, ns: not significant). n = 45 per group.

The mRNA levels of *dZIP71B* were significantly increased in the parents (Figure 6a), while the male and female offspring had significant (*p* < 0.05) reduction in *dZIP71B* mRNA compared to control (Figure 6b).

In addition, there was a significant (*p* < 0.05) reduction in fold change in *dZnT35C* mRNA in both the male and female parents (Figure 6c) as well as the male and female offspring compared to control (Figure 6d).

### 3.7. Effects of Parental Zinc Deficiency on DILP2 and dPEPCK mRNA Levels

**Figure 7:**
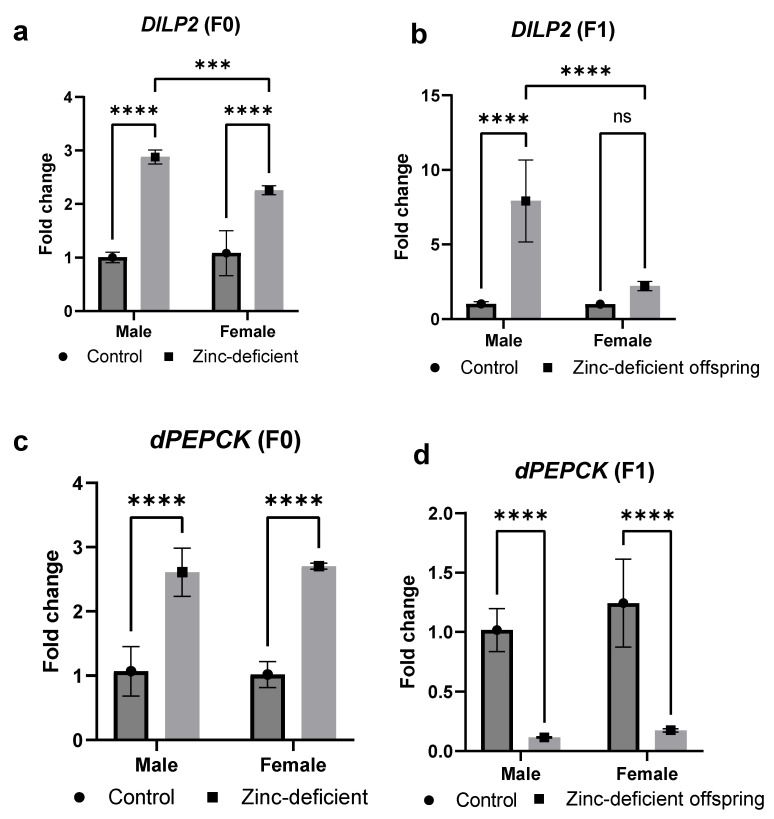
Effects of parental zinc deficiency on *DILP2* (**a**,**b**) and *dPEPCK* (**c**,**d**) mRNA of flies. Bars represent mean ± SD. Data were analyzed using two-way ANOVA followed by Bonferroni’s post hoc test. ns: not significant. Asterisks represent significant difference at varying *p* values (***: 0.0002, ****: <0.0001, ns: not significant). n = 45 per group.

The mRNA levels of *DILP2* were significantly (*p* < 0.05) increased in both zinc-deficient parents and offspring (Figure 7a,b). However, *dPEPCK* mRNA was significantly (*p* < 0.05) increased in the parents (Figure 7c) but decreased in the offspring (Figure 7d) compared to control.

### 3.8. Effects of Parental Zinc Deficiency on SOD1 and CAT mRNA Levels

**Figure 8:**
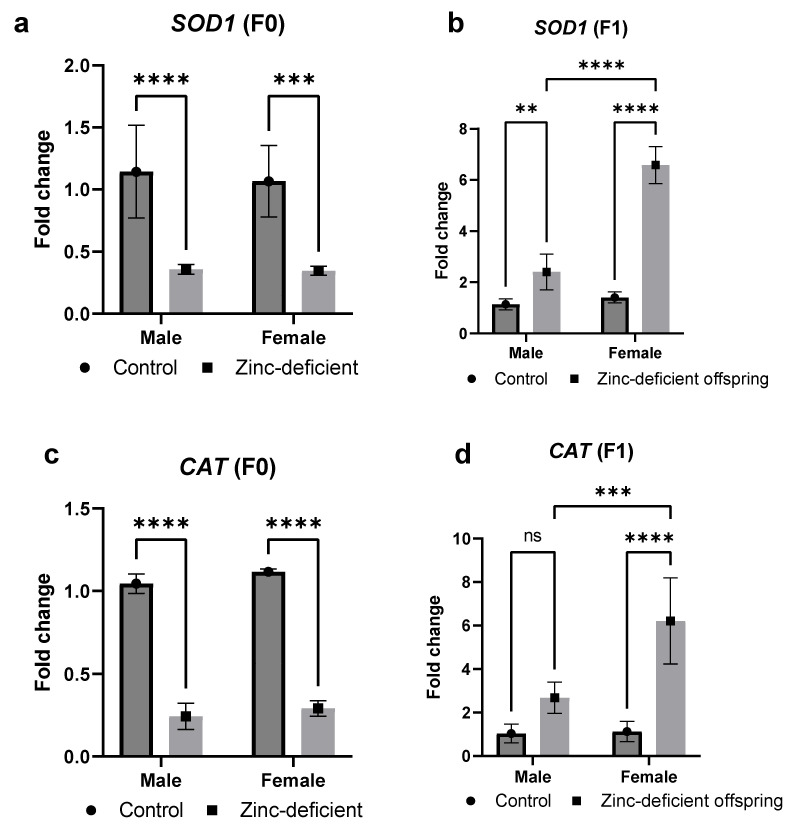
Effects of parental zinc deficiency on *SOD1* (**a**,**b**) and *CAT* (**c**,**d**) mRNA of flies. Bars represent mean ± SD. Data were analyzed using two-way ANOVA followed by Bonferroni’s post hoc test. ns: not significant. Asterisks represent significant difference at varying *p* values (**: 0.0021, ***: 0.0002, ****: <0.0001, ns: not significant). n = 45 per group.

There was a significant (*p* < 0.05) decrease in *SOD1* mRNA in the zinc-deficient parents (Figure 8a) while a significant (*p* < 0.05) increase in the *SOD1* mRNA was observed in the offspring (Figure 8b) compared to control. Similarly, the fold change in *CAT* mRNA was significantly (*p* < 0.05) reduced in the parents (Figure 8c) but increased in the offspring (Figure 8d) compared to control.

### 3.9. Effects of Parental Zinc Deficiency on EGR and UPD2 mRNA

**Figure 9:**
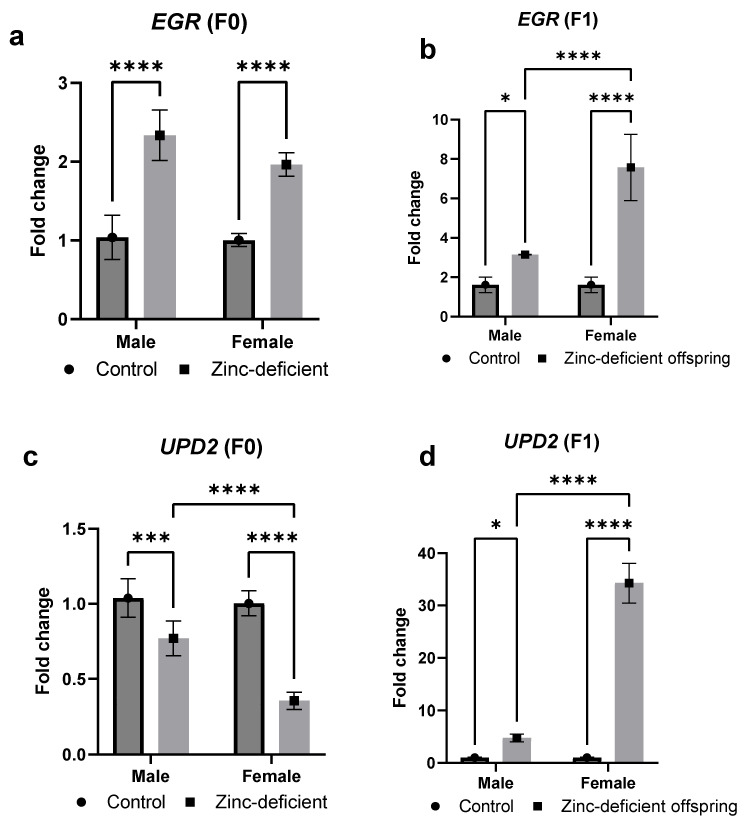
Effects of parental zinc deficiency on *EGR* (**a**,**b**) and *UPD2* (**c**,**d**) mRNA of flies. Bars represent mean ± SD. Data were analyzed using two-way ANOVA followed by Bonferroni’s post hoc test. ns: not significant. Asterisks represent significant difference at varying *p* values (*: 0.0332, ***: 0.0002, ****: <0.0001). n = 45 per group.

There was a significant (*p* < 0.05) increase in the fold change in *EGR* mRNA in both the zinc-deficient parents (Figure 9a) as well as the male and female offspring (Figure 9b) compared to control. However, the expression of *UPD2* mRNA was significantly (*p* < 0.05) reduced in the parents (Figure 9c) but increased in both the male and female offspring (Figure 9d) compared to control.

## 4. Discussion

We have demonstrated gender-specific responses to zinc deficiency in this study, providing insights into the interplay between prenatal zinc status and offspring’s metabolic health. Moreover, prenatal nutrition is an important determinant of an offspring’s adult life risk of disease [9,10]. Particularly, zinc is an essential element for a healthy pregnancy [10] and has been shown to regulate various metabolic functions through its effects on neurotransmitters, enzymes, and hormones [11]. Also, maternal zinc deficiency has been reported to cause adverse pregnancy outcomes [6,10]. Thus, to understand the implications of zinc deficiency in both parents, we investigated the combined effects of maternal and paternal zinc deficiencies.

To make sense of the zinc level changes we observed, we studied the expression of zinc transporters in the offspring responsible for both zinc absorption and excretion and observed changes that suggested compensatory mechanisms to boost zinc bioavailability. *dZIP1* facilitates the transfer of zinc from the extracellular matrix into the cytosol for dietary zinc absorption in the enterocytes of the midgut [12], while *dZnT1* facilitates its transport out of the enterocytes across the basolateral membrane [13]. Increased *dZIP1* expression in the zinc-deficient parents could have been an adaptive response to enhance zinc absorption, while the contrasting male and female offspring responses (decrease in males and increase in females) suggest gender-specific differences in the regulation of zinc transporters. Similarly, *dZnT1* expression changes suggest enhanced absorption especially in the male zinc-deficient parent and male offspring. In this case, also, gender-specific effects were visible with decreased expression in both female zinc-deficient parent and female offspring. Furthermore, *dZip71B* located in the drosophila Malpighian tubules facilitates the import of zinc from the circulation into the tubular cell [14], and together with *dZnT35C* excrete zinc from the tubular cell. The increased dZip71B levels in the zinc-deficient parents suggest a reabsorption mechanism, likely in response to reduced zinc levels, while reduced *dZnT35C* expression may have favored decreased zinc export to conserve the limited zinc available. Moreover, decreases in *dZip71B* and *dZnT35C* mRNA levels in the offspring may have been adaptive responses to conserve zinc by reducing its excretion.

Because we observed changes in zinc levels and expression of zinc transporters, and bearing in mind that zinc homeostasis is important for metabolic function [11,15,16], we evaluated select metabolic indices. Accordingly, the increases in glucose levels in both male and female parents suggested a potential association between parental zinc deficiency and altered glucose regulation, as reported previously [10,15]. Although the offspring’s glucose levels did not change in response to zinc deficiency, the increases in trehalose levels in the parents and offspring suggest altered carbohydrate metabolism, since trehalose is one of the main circulating forms of glucose in drosophila [17]. Thus, elevated trehalose levels indicate altered sugar metabolism in response to zinc deficiency. Glycogen is also a major storage form of glucose and an important source of biofuel in drosophila [17,18]. Therefore, the reduced levels in both parents and offspring further support dysregulated glucose metabolism. Lipid changes were also assessed since zinc homeostasis is known to affect lipid levels [19]. The reduction in triglyceride levels further suggests zinc-induced altered lipid metabolism.

To understand the basis of the metabolic changes observed, we studied the mRNA changes of select genes related to glucose metabolism. Accordingly, elevated *DILP2* mRNA in both zinc-deficient parents and their offspring suggest the zinc-induced dysregulation of *DILP2*, which plays a pivotal role in insulin signaling [20]. Also, *PEPCK* is a key player in the gluconeogenic production of glucose in drosophila [21], and its zinc-induced increases in the parents suggest the stimulation of gluconeogenesis with consequent increases in glucose levels and/or its storage forms of trehalose or glycogen as seen in this study. Unexpectedly, however, decreased offspring *dPEPCK* mRNA levels may imply compensatory mechanisms and further highlight the complexity of the metabolic response to zinc deficiency.

Since zinc homeostasis has been shown to influence inflammation and redox status [16], we also evaluated the changes in inflammatory and antioxidant markers and mRNA levels of some select genes. As expected, catalase activity and total antioxidant capacity were reduced in the zinc-deficient parents [16,22]. Interestingly, compensatory mechanisms may have induced unexpected changes in antioxidant responses in the offspring. However, the elevated levels of malondialdehyde, a marker of lipid peroxidation [23], in both zinc-deficient parents and offspring suggest redox imbalance due to parental zinc deficiency. Similarly, the expression of *EGR*, a drosophila homologue of tumor necrosis factor, increased in response to zinc deficiency indicating a proinflammatory state [24]. Moreover, the expression of another proinflammatory marker, *UPD2*, known to be involved in the regulation of insulin signaling [25], was increased in the offspring although it was decreased in the parents. Overall, these changes in lipid, glucose-related, redox, and inflammatory markers, in addition to indicating zinc-induced metabolic dysregulation, further suggest the complex regulatory mechanisms in response to zinc deficiency.

## 5. Conclusions

This study sheds light on the intergenerational impact of both maternal and paternal zinc deficiencies on various metabolic outcomes. The observed alterations in body weight, zinc levels, glucose metabolism, lipid homeostasis, antioxidant enzyme activities, and gene expression profiles underscore the significance of zinc in maintaining overall health. Moreover, the significant changes in zinc transporter genes, metabolic markers, and inflammatory factors provide valuable insights into the molecular mechanisms underlying the effects of zinc deficiency. Gender-specific responses and inheritance pattern variations highlight the complexity of the interplay between parental zinc status and offspring outcomes. Overall, this research contributes significantly to our understanding of how zinc influences metabolic, antioxidant, and inflammatory pathways across generations. The insights from this Drosophila model pave the way for future investigations in mammals, including humans, potentially unveiling broader implications for our health and well-being.

## 6. Limitation

While this study employed established protocols for sample preparation using whole-body homogenates [8], this approach has limitations. Homogenization offers several advantages, including simplicity and efficiency, particularly when analyzing large numbers of flies. However, when used on Drosophila, it reflects a combined picture of hemolymph and cellular content, potentially providing a broader representation of the organism’s overall metabolic and redox status. Thus, the results of this study should be interpreted within the context of this limitation. Similarly, studies aiming for more precise measurements of hemolymph metabolic markers may benefit from alternative sample preparation techniques, such as microcapillary aspiration.

## Figures and Tables

**Table 1:** Primer sequences for genes of interest.

S/N	Gene	Primer	Sequence	Annealing Temperature (°C) *
1	*dZIP1*	Forward	AGGCTCAACAACCCTACTTTC	60
		Reverse	TTACCACCCTTGTGTGTTTCT	
2	*dZnT1*	Forward	CACCATTCAGCCAGAGTTCA	60
		Reverse	CTTCTTCCGTGGTAGGACAATC	
3	*dZip71B*	Forward	CCCAGTAGCCTTCATGGTAATC	62
		Reverse	GCAAAGGCGGTAGCAAATC	
4	*dZnT35C*	Forward	GTGTTGTAACGTGTGGTGTTAG	62
		Reverse	CGTTTGGCAATCGGTGTATC	
5	*DILP2*	Forward	GTACTCAATTCCCTGGCTGAA	55
		Reverse	CGCAGAGCCTTCATATCACA	
6	*PEPCK*	Forward	TCAATGGCGAATCCTGCTAC	60
		Reverse	CTTCACGTCCACCTTATCCTTC	
7	*SOD1*	Forward	CGGTCACACCATAGAAGATACC	65
		Reverse	CAGACAGCTTTAACCACCATTTC	
8	*CAT*	Forward	TGGTCGTCTGTTCTCCTACT	65
		Reverse	CCGCTGGAAGTTCTCAATCT	
9	*UPD2*	Forward	TTGACCATAAACGCCTCCTATC	60
		Reverse	GTGAAAGTTGAGACGCTCCT	
10	*EGR*	Forward	TGAGGCAACTTCCAAAGAGAG	60
		Reverse	CGGATCTGGCTGAAAGAAGAG	
11	*RPL32*	Forward	GGATCGATTCCTGTGAGAGTTC	60
		Reverse	TGGGCAGTATCCATTGAGTTT	

* PCR conditions: reverse transcription at 45 °C for 5 min; pre-denaturation at 94 °C for 30 s; 40 cycles of denaturation at 94 °C for 5 s, annealing at the corresponding temperature for 15 s, and extension at 72 °C for 10 s. CAT: catalase; dILP-2: Drosophila Insulin-like peptide-2; dZIP1: Drosophila Zrt-, Irt-like Protein 1; dZip71B: Drosophila Zrt-, Irt-like Protein 71B; dZnT1: Drosophila Zinc Transporter 1; dZnT35C: Drosophila Zinc Transporter 35C; EGR: Eiger; PEPCK: phosphoenolpyruvate carboxykinase; RPL-32-60S: ribosomal protein large subnunit-32; SOD1: superoxide dismutase 1; and UPD2: unpaired 2.

## Data Availability

The datasets generated during and/or analyzed during the current study are presented in this paper.
